# Conceptual unclarity about COVID-19 ethnic disparities in Sweden: Implications for public health policy

**DOI:** 10.1177/13634593221074866

**Published:** 2022-02-13

**Authors:** Anna Bredström, Shai Mulinari

**Affiliations:** Linköping University, Sweden; Lund University, Sweden

**Keywords:** COVID-19, discourse, ethnicity, race, Sweden

## Abstract

The COVID-19 pandemic has shed light on abundant racial and ethnic health disparities in many countries around the world. In Sweden, statistics on COVID-19 mortality and morbidity from both the first and the second wave of the pandemic show that foreign-born individuals have been disproportionately affected, compared to Swedish-born individuals. However, as demonstrated in this article, key stakeholders including politicians, public authorities, mainstream media, and medical researchers do not draw on the same explanatory framework when conceptualizing the health disparity. Probing the different discourses that were articulated through oral and written accounts during the first wave, the article identifies three different frameworks of how ethnic health disparities in relation to COVID-19 were understood in Sweden: the socioeconomic framework, the culturalist framework and the biological framework. We discuss the importance of our findings for health policy and argue for continued interrogation of epidemiological knowledge production from a critical vantage point in order to successfully combat health inequalities.

## Introduction

As has been the case in many countries, epidemiologic data in Sweden show evidence of ethnic health disparities in relation to COVID-19 (Folkhälsomyndigheten, 2020a). In an analysis of national, individual-level data concerning people above the age of 20 between 13 March and 7 May 2020, Drefahl et al. (2020: 3) demonstrate that “immigrants from low- and middle-income countries are approximately twice as likely to die, as compared to individuals born in Sweden.” They also point out that the disparity applies for people of both working age and retirement age, and for women and men.

These findings from Sweden resemble studies of ethnic and racial disparities in COVID-19 morbidity and mortality from other countries. Data published in June 2020 from Public Health England (2020: 6), for instance, showed that “death rates were highest among people of Black and Asian ethnic groups,” and that “after accounting for the effect of sex, age, deprivation and region, people of Bangladeshi ethnicity had around twice the risk of death when compared to people of White British ethnicity” (Public Health England, 2020: 39). Similarly, the US Centers for Disease Control and Prevention’s Morbidity and Mortality Weekly Report has repeatedly reported that members of minority racial and ethnic groups are disproportionately represented in the statistics of COVID-19-associated death at both national and state levels (e.g. Gold et al., 2020; Podewils et al., 2020; Wortham et al., 2020). Racial and ethnic minority healthcare staff have also been reported to be at higher risk of COVID-19 morbidity and mortality than their colleagues (Pan et al., 2020; Public Health England, 2020).

Ethnic and racial disparities in relation to COVID-19 have led to a “call for action” amongst many scholars and organizations engaged in equal health (Bhala et al., 2020; Laurencin and McClinton, 2020). Coming to term with these inequalities, they have argued, is necessary in order to curb the rapid spread of the coronavirus. The pandemic is also said to shed light on already existing racial and ethnic health disparities. The Association for Black Cardiologists in the United States has for instance pointed to how the pandemic “presents an opportunity to decisively address race-based or ethnicity-based inequalities that undermine cardiovascular health” (Chin-Hong et al., 2020: 3).

Amongst these researchers and experts, a common explanation as to why some groups are more vulnerable to the virus stems from a social determinants of health perspective. Some of the key medical conditions that have been identified as risk factors for severe COVID-19, such as hypertension, obesity and type 2 diabetes, are intimately related to the living conditions and lifestyles that come with poverty (Golestaneh et al., 2020; Patel et al., 2020). Overcrowding and intergenerational housing are also emphasized in this research, as are the facts that people of poorer socioeconomic conditions rely on public transport to a larger extent and are more exposed at work. Many are essential workers, employed in sectors such as care, the food industry and transport – jobs that cannot be done from home and where physical distancing can be difficult to implement.

In addition to these socioeconomic perspectives, some scholars point to structural racism as an important aspect to consider. One example is how comorbidities among racial and ethnic minorities may go undetected as some minorities are less inclined to seek healthcare or are not cared for to the same extent by the healthcare staff. Kirksey et al. (2021) write for instance that bias and stereotypes among (white) healthcare workers seemingly affect the treatment and care of non-white patients and that this is particularly evident at times when healthcare systems are under heavy pressure. Relatedly, Khazanchi et al. (2020) point to how poverty and lack of targeted information together with structural racism affect health-seeking behaviors. Another example of structural racism refers to a purportedly neutral algorithm that was used to prioritize between patients and resources in the United States. However, it was subsequently revealed that the algorithm was built on pre-existing data about different groups’ health status, black and ethnic minority patients ended up being less prioritized for receiving ventilators (Williams et al., 2020). Finally, it has been suggested that biological factors contribute to the observed association of severe illness and ethnic/racial minorities, in particular vitamin D deficiency (Liu et al., 2021).

That research on racial and ethnic disparities relating to COVID-19 draws upon different discourses – for example socio-economics, structural racism, biological differences – points to possible tensions and instabilities in the knowledge produced in this field. Indeed, tensions and instabilities seem to permeate ethnic and racial categorization at its very basic level; the frequent use of BAME (black, Asian, and minority ethnic) in the discussions on COVID-19 health disparities in the UK, has for instance, been criticized for “indiscriminately combin[ing] people from different geographical, behavioral, social, and cultural backgrounds” (Khunti et al., 2020: 1). In this article we are interested in further interrogating the knowledge production surrounding racial and ethnic disparities in relation to COVID-19, using the example of the Swedish public health debate. Our focus is on the discourses, or, as we call them in this article, “explanatory frameworks” that key stakeholders draw upon when they make sense of why some groups have been more or less severely affected. With reference to Michel Foucault and Nikolas Rose, Vallgårda (2011: 7) encourages public health scholars to study the “problem of problematization” as “a discursive process whereby issues are framed and thereby made accessible to political action.” In a similar vein, Shim (2002) urges us to explore how “ideas about difference” are constructed in epidemiological knowledge production. Applying concepts from science and technology studies such as “black box” and “boundary objects,” Shim shows how both the production of differences and inherent instabilities and contradictions within epidemiological knowledge production are silenced. This article will contribute knowledge along this line of inquiry and will show how three explanatory frameworks were present in the research and debate on ethnicity and COVID-19 in Sweden: a socioeconomic framework, a culturalist framework and a biological framework. In terms of method, we build on a systematic reading of expert and media reports, oral statements and published research from key stakeholders in Sweden. These stakeholders include the Swedish government and involved authorities, in particular the Public Health Agency of Sweden which has played a crucial role in the pandemic response, but also key politicians from various political parties including the right-wing opposition parties, mainstream media, medical professionals and researchers. Another key stakeholder is of course the concerned patients. While their first-hand voices are lacking in this article, some of the analyzed media coverage and debate articles do include patients’ and close relatives’ experiences and points of view.

The empirical material covers the period from early-March to mid-September 2020 (i.e. the first pandemic wave), and contains transcriptions of the joint press conference held all weekdays until early June, and subsequently twice weekly, by the Public Health Agency of Sweden, the National Board of Health and Welfare and the Swedish Civil Contingency Agency (*n* = 95). Our empirical material also includes published reports and commentaries from the Public Health Agency and other authorities (*n* = 10); published media articles (*n* = 42) from the largest daily newspapers *Dagens Nyheter, Svenska Dagbladet* and *Sydsvenskan*, and evening press *Aftonbladet* and *Expressen*; and the journal of the Swedish Medical Association, *Läkartidningen*, covering the same time period (*n* = 6). The articles in both daily and evening press, and in the Medical Association journal, were identified using the following search words (in Swedish): COVID-19, corona, foreign-born, ethnicity, migration, immigrant, genetics, Somali. Using the same search words with the addition of “Sweden,” we also searched the Linköping university library databases for published research articles (*n* = 10).

The material was subsequently coded following principles for discourse analysis (Winther Jørgensen and Phillips, 2002) focusing on the production of meaning, intertextual and interdiscursive linkages between different contexts and representations of group identities. More specifically, we departed from the following research questions to capture the main structure of the discourses – the explanatory frameworks – that took shape in the empirical material: How is the ethnic health disparity described and explained in different contexts and among different stakeholders? What words are used and what do they indicate? How is race/ethnicity conceptualized? What patterns emerge in the material as a whole?

Below we give a brief overview of ethnic disparities in relation to COVID-19 in Sweden under the first wave, followed by our analysis of the present discourses divided in three sections with headings that capture the explanatory frameworks that our analysis revealed: *the socioeconomic framework; the culturalist framework* and *the biological framework*. The article ends with a concluding discussion of our findings in relation to health policy where we argue for the importance of being attentive both to policy outcomes that may follow upon a particular explanatory framework, and to instabilities and contradictions within public health knowledge production.

## Background: Ethnic disparities in relation to COVID-19 in Sweden

The corona pandemic has put Sweden in the limelight mainly for having less restrictive measures than many other European countries. The Swedish authorities have, for instance, been reluctant to impose strict lockdowns with the argument that measures need to be sustainable over time and that lockdowns in general – and the closing of schools in particular – have huge negative effects on public health at large (Olofsson et al., 2021). The Swedish strategy has been misrepresented by international media as ignorant and completely lax (Irwin, 2020). However, extensive measures to curb the virus have been issued also in Sweden, including online education for youth and young adults, the closing of many public institutions, and a limit of eight people for social or public gatherings (krisinformation.se 2020; Zeiler, forthcoming).

In this article, however, it is not the overall “Swedish strategy” that is of primary interest, but, much more restrictively, how COVID-19 ethnic disparities were understood. Sweden is an interesting case in this regard too. It is a country with a long history of being a universal welfare state with strong emphasis on equality (Schierup et al., 2006). It is also a country with extensive and high-quality population registries, which are used in public health and medical research, and a relatively large population with migrant or minority background (Schierup et al., 2006). In Sweden the concept of race is officially repudiated with reference to the history of racial biology and eugenics (SOU, 2015: 103), and instead, concepts of “migrants” and “migrant background” are used as proxy for race/ethnicity in public discourse, although population statistics, as will be discussed below, refer only to country of birth.

That foreign-born individuals in Sweden were particularly vulnerable to the virus was acknowledged early on in the pandemic. Sweden had its first confirmed SARS-CoV-2 related death on March 11, and already on March 24 came the first alarming reports about ethnic disparities. It was a representative of the Swedish-Somali medical society who, when interviewed by the public service company Swedish Television, revealed that of the then 15 deaths linked to SARS-CoV-2 in Stockholm County as many as six patients were of Somali origin. This apparent overrepresentation was subsequently reported by other major news sites, and about a week later – at a press conference on 2 April – a journalist posed the question if the authorities should include country of birth in its epidemiological reports from now on. State epidemiologist Anders Tegnell from the Public Health Agency answered that, at this point in time, such information would risk revealing the identity of the patients and would be unethical. However, Tegnell confirmed that the Agency did see the importance of paying close attention to patient demography, and 12 days later, at the press conference on 14 April, he presented some initial statistics that pointed to overrepresentation of severe infections among foreign-born individuals.

In mid-June, the Agency published a full report that for the first time described the demography of patients with confirmed SARS-CoV-2 in Sweden (Folkhälsomyndigheten, 2020a). The report verified the initial signals of Somalis being overrepresented among those who had died with COVID-19. The report measured incidence (number of confirmed cases per 100,000) and mortality between 13 March and 7 May. Over this period, Sweden still primarily tested those who had to seek hospital care; therefore, a high incidence pointed to high morbidity as those with mild infections did not seek hospital care.

The report showed that in addition to Somalis, people born in Turkey, Ethiopia, Chile and Iraq had significantly higher incidence than people born in Sweden. When it came to fatality, people born in Finland were the worst affected group, most likely due to the age structure of Finnish migrants. The statistics also showed that vulnerability shifted over time: Somalis had the highest numbers in the beginning of the period, but were superseded by Iraqis toward the end. The shift corresponds to the description of cluster virus outbreaks, and points to the necessity of understanding statistics like these as a temporary snapshot.

The statistics were based on country of birth and concern people who reside in Sweden. The report showed a national picture, thus there might be regional differences that the report did not capture. Official population statistics produced by Statistics Sweden (SCB) concern people registered in Sweden and do not include, for instance, asylum seekers. Country of birth is the main category used by Statistics Sweden and a person born outside of Sweden is registered as foreign born regardless of parents’ country of birth or citizenship. Country of birth is recognized as a rather limited category as it does not capture if the person in question belongs to an ethnic minority. Thus, the category “born in Turkey” may include both Kurdish and Assyrian minorities. Nor does country of birth capture “second generation migrants,” which could also be relevant in this case as all of the above-mentioned countries have had migration to Sweden for decades.

Despite these limitations, the numbers in the report indicated major disparities. People born in Turkey had, for instance, an incidence of 753 per 100,000, compared to Swedish-born with an incidence of 189 per 100,000. This disparity has subsequently been confirmed by other studies (Drefahl et al., 2020; Hanson et al., 2020; Socialstyrelsen, 2020) and the ethnic health disparity also mirrors, as mentioned earlier, research on other countries.

## The socioeconomic framework

In general, the key actors responsible for managing the pandemic in Sweden emphasized socioeconomic aspects as well as possible confounders. The above-mentioned report on the country of birth of COVID-19 patients was presented by state epidemiologist Tegnell at the press conference on 18June. He introduced the report by saying that “we have talked about this before, that we already early on in the outbreak saw that people born in other countries than Sweden, but living here, had a very high morbidity in the beginning, they ended up in intensive care units and even death was more common in these groups than among those born in Sweden.”

It is clear, Tegnell continued, that country of birth *does* make a difference when it comes to increased risk, and he explained that the Agency would continue its research with more in-depth studies. Tegnell was reluctant to present more than a descriptive account, but he nevertheless mentioned that there were “many factors” that may play a role when it comes to explaining the difference. He pointed specifically to “socio-economics, life conditions and underlying illnesses.” Later on, at the same press conference, after being probed by a reporter from Swedish Television (the national public broadcaster), he also mentioned “overcrowding” and “poor working conditions” as possible reasons behind the disparity.

Like Tegnell, the key actors have been reasoning in the following way: poor living conditions make some populations more exposed to the virus, that is, more affected populations live in more densely populated areas and in smaller apartments, and they have no alternative but to use public transport. Their vulnerability is also enhanced by the fact that the same group of people are exposed to the virus at work; many of them are, for instance, taxi/bus drivers, cleaners, and care workers (Folkhälsomyndigheten, 2020b). There are also several examples of research that largely backs the socioeconomic explanations put forward by the public authorities. A study by Hanson et al. (2020) looked at excess mortality between the period of February and May 2020 as a way to investigate if, and to what extent, country of birth affected COVID-19 mortality. Hansson et al. showed that people born in Syria, Somalia and Iraq had a much higher excess mortality than people born in Sweden, the Nordic countries, the EU and North America. The authors pointed out that it is the same groups – people born in Iraq, Somalia and Syria – that are the least established in Swedish society, many are for instance relatively newly arrived refugees, and conclude that: “our hypothesis is that the virus has been transmitted in circles, between service and care professions, commuting to and from work and [the urban districts] where people live” (Hanson et al., 2020: 2).

While agreeing to the socioeconomic framework, scholars such as Hansson et al. nevertheless criticized the Public Health Agency for not reaching the most vulnerable in its pandemic strategy. Communication flaws, as well as a lack of attention to the different conditions in which people live and work, are pointed out as failures of the Swedish approach that they see as appealing only to a homogenous majority population. The need of specific information on how to practice physical distancing when living together across generations has also been suggested (Hanson et al., 2020; Jakobsson et al., 2020).

Some journalists and opinion-makers also raised concerns along these lines. In a series of opinion articles in the daily press *Svenska Dagbladet* (2020-04-10, 2020-05-12) journalist Nuri Kino described how COVID-19 hit his Assyrian community. During the first 3 weeks of the epidemic, 43 of the 225 that had died in Stockholm were Syrian/Assyrians. “I know many of them myself,” he writes, “or their children or their relatives,” and goes on to tell how he decided to isolate himself to take care of his old mother who suffers multiple illnesses. Under normal circumstances, the mother would be looked after by the home care services, but with corona, home care staff became a liability for Kino.

## The culturalist framework

However, at the same time as evening and daily press reported research and opinions that featured socioeconomic perspectives, mainstream media also presented a slightly different view. For one, media “gave voice” to the concerned groups either through “representatives” such as Nuro Kino and others, or through coverages focusing on the experiences of for instance Assyrians and Somalis in Sweden. These media stories focused on stigma and “blame-the victim” attitudes of Swedish society. The critique was not directed to the concerned authorities as such, but to society at large. Nuri Kino mentions, for instance, that Syrian/Assyrian doctors who were friends of his met a “blame the victim” attitude among the healthcare staff in the hospitals where they worked. Similarly, a long coverage in the Daily press *Dagens Nyheter* (2020-05-02), based on interviews with Somali community representatives from one of the suburbs of Stockholm that was hit hard by the virus, had the telling title: “How the Swedish-Somalis were blamed for their own death.” The story focused on the intense vulnerability that the Somali community experienced as they were hit unprepared early on in the pandemic. They had no chance to protect themselves or their loved ones, and they had to continue to work, commute and take care of their elders. And just as Kino, the Somali interviewees experienced an attitude among “Swedes” that the Somalis had themselves to blame. They argued that Swedish society met the Somali community with skepticism, saying that they (the Somalis) lacked knowledge and were poor receivers of information due to analphabetism and traditional cultural and religious practices.

What the Swedish-Somali gave witness to was a discourse where *cultural differences* were portrayed in a stereotypical and derogatory way. We call this discourse the culturalist framework, and mainstream media also partook in spreading this framework. During the spring of 2020, the culturalist framework was primarily represented by some key politicians of center-right and right-wing parties in Sweden. One of them was the leader of the Swedish Christian Democrats (which is more right-wing than many of its Western-European counterparts), Ebba Busch. On 2 April she wrote a debate article in the evening paper *Aftonbladet*, entitled “Dare to speak clearly about the corona and the suburbs.” The article starts with her stating that “the disaster has already happened,” pointing to the immense spread of SARS-CoV-2 in the “suburbs.” She also states that there are many reasons behind this development, including “crowded living,” but there may also be “culture-specific causes.” Among other things she refers to how “Somalis have not the same tradition as do Swedes when it comes to written information and medical practices.” She also states that the Swedish strategy rests upon norms that may apply to people who are born and raised in Sweden. In the “suburbs,” many are born in another country and “most in culturally remote societies,” she argues.

Busch was criticized for this article. Representatives of the Somali community, for instance, pointed out how her argument partakes in increasing racism and discrimination (*Aftonbladet* 2020-04-03). However, some other actors, primarily other center-right politicians and opinion-makers, supported her views, or contributed to the same culturalist framework by proposing, for instance, that the many deaths in elderly care were somehow linked to care staff with foreign backgrounds with insufficient competence in the Swedish language (Sabuni et al. in *Sydsvenskan* 2020-06-11), or that the Swedish strategy is too “soft” and therefore does not appeal to people with other cultural backgrounds (Mahmood in *Dagens Nyheter* 2020-04-15).

## The biological framework

As has been described above, there were thus two explanatory frameworks that focused on the socioeconomic and cultural differences between Swedes and some “foreign-born” groups, respectively. There is more to be said about the two discourses, but before we do that, we want to highlight a third, alternative framework that also frequented the discussion on ethnic disparities in relation to COVID-19 during this period. This framework emphasized underlying biological susceptibilities.

This “biological framework” was mainly present in the medical literature. In the media discourse and the materials from the public authorities, biological differences were only mentioned in passing. In one of his media coverages, for instance, Nuro Kino mentions that he had heard of underlying genetic causes that would explain why Syrian/Assyrian suffered so badly (*Svenska Dagbladet* 2020-04-10). Ideas about genetic differences also surfaced at the press conferences (e.g. 2020-05-08), mostly as a question from a journalist who sought to find alternative explanations for why some groups seemed to be worse off, but was bypassed by the representative of the Public Health Agency who remained firm with the socioeconomic framework.

In the medical literature, however, ideas of biological/genetic differences between different ethnic or racial groups were more frequent, but far from the only discourse present. On the contrary, much medical research stressed the socioeconomic argument as well. Still, regarding biological susceptibilities, various hypotheses were discussed. For example, evidence of vitamin D as protection against severe COVID-19 appeared as an argument in the medical professional journal *Läkartidningen*. As vitamin D deficiency is common among some racial/ethnic groups, the authors argued, referring to people of “African descent” as well as other nationalities and ethnic groups (“Iraqi” and “Syrian” descent in Sweden, “Asian” descent in the US), it could be that vitamin D levels can explain the vulnerability among some foreign-born Swedes (Humble et al., 2020). In this particular article the authors also questioned the socioeconomic framework and argued that socioeconomics cannot be seen as the sole explanation of the ethnic disparity of COVID-19 seen in many western countries, indicating that vitamin D deficiency may be a complementary or better explanation.

Another example of the “biological framework” is to be found in population genetics research that maps genetic variation within and between populations. In relation to the pandemic, *the COVID-19 host genetics initiative* (an international consortium that seeks to generate, share and analyse data to learn about the genetic determinants of COVID-19 susceptibility, severity and outcomes) identified a link between a particular genetic variant and severe COVID-19. The same genetic variant was subsequently recognized by the Swedish geneticists Zeberg’s and Pääbo’s (2020) (the latter a well-known director at the Max Planck Institute in Leipzig, Germany) as “Neanderthal DNA,” known from Pääbo’s earlier studies.

Linking the identified genetic variant that may explain severe COVID-19 to Neanderthal DNA, Pääbo and Zeberg argue, may be useful in order to understand ethnic disparities as this specific genetic variant is highly present in South Asia, particularly in Bangladesh – and Bangladeshi migrants have been identified as particularly vulnerable in the British context. The study was reported worldwide, but was only mentioned in the evening press *Expressen* (2020-07-05) in Sweden during the time-frame set for this study.

## Concluding discussion

What we have seen thus far is three quite distinct explanatory frameworks put forward by different stakeholders. Needless to say, there are also overlaps between the frameworks, as for instance when Hanson et al. (2020) argue that social and cultural aspects explain the high incidence among some groups, whereas biology explains the mortality differences. Similarly, international research on COVID-19 disparities sometimes defines ethnicity as including “genetic make-up” as well as “social/cultural identity and behavioral patterns” (Pan et al., 2020: 2). However, rarely is such complexity fully fleshed out in the discussion. In the case of Hanson et al. (2020), for instance, their main emphasis remained on the structural constraints related to housing and work, consistent with the socioeconomic framework.

Moreover, as our analysis is primarily conceptual, we have focused on *the ways of reasoning* rather than the frequency of a particular argument. However of the three discourses present, the culturalist argument echoes the prevailing discourse on migration and migrant integration in Sweden, and in Europe at large. In the mainstream public debate, ethnicity has been conflated with culture ever since the decline of the racial biology paradigm after World War II (Schierup et al., 2006). In the 1980s, culture was articulated within the then dominant model of multiculturalism cherishing cultural differences and pluralism. Since the turn of the millenium, however, the culturalist framework has become more exclusionary and neo-assimilatory in its outlook, portraying migrant cultures as containing dubious values that are incompatible with “Swedish” norms of equality, liberalism and modernity (Bredström and Bolander, 2018; Bredström, 2008).

In comparison to the mainstream public debate, the strong presence of socioeconomic perspectives makes the discussion on ethnic health disparities in relation to COVID-19 somewhat unique. This may be explained by the specific epistemic context of the pandemic: specifically, the key actors stewarding the Swedish pandemic response belong to a public health “thought community” (Fleck, 1979) where there is a strong belief in the social determinants of health (Vallgårda, 2007). Indeed, characteristic for the Swedish corona strategy has been its emphasis on *public health* rather than a narrower focus on infectious disease epidemiology. Every restriction has thus been evaluated from a broader public health perspective. The general well-being of children and youth has for instance been put forward as an important argument for keeping elementary schools open and letting leisure activities for youth continue with limited restrictions.

The emphasis on socioeconomics may also be interpreted as a more progressive outlook on migration and ethnicity as compared to the mainstream culturalist framework. However, letting socioeconomic factors be the sole explanation for ethnic health disparities would not suffice as it would reduce race/ethnicity to class. Most importantly, it would conceal how racism may contribute to inequality in health. As mentioned in the introduction, there is some international research that points to how racist structures in society affect health outcomes for COVID-19-patients (Khazanchi et al., 2020; Kirksey et al., 2021). To what extent structural and institutionalized racism could explain the disparities in mortality and morbidity in Sweden seems however to be a blind spot in the Swedish debate, and racism was not identified as an explanatory framework in the material under study here, apart from the media reports on stigma and “blame the victim” attitudes in general society.

Finally, while both the socioeconomic and culturalist frameworks are familiar to discussions around ethnicity in Sweden, the third framework’s notion of biological differences are less so. In the Swedish context, notions of racial differences are, as mentioned, officially refuted, yet ideas about biological differences have found a way back through medicine also in Sweden (Mulinari et al., 2021). This development has its epistemic roots within biomedicine, primarily studies of genetic variation among and between populations. The ways in which race and ethnicity is used as proxy for populations in genetic variation studies have been subjected to much criticism internationally both within medicine and social science (e.g. Cooper et al., 2003; Roberts, 2011). Critics argue that it has even led to an ontological shift whereby the *social* categories of race and ethnicity are being redefined as *biological* categories. Bliss (2015) shows for instance that in international health policy, health disparities were originally addressed from a social justice perspective. In the past two decades, largely as a consequence of genomic difference studies, health disparities are increasingly understood and targeted from within a biomedical viewpoint.

It is too early to tell to what extent the attention to biological differences in relation to ethnic disparities of COVID-19 mortality, morbidity and vulnerability will lead to a more biological understanding of ethnicity in Sweden. The very existence of a biological framework is still worth noticing, not least as it may affect policy interventions. That medical experts recommend vitamin D supplements for racial and ethnic minorities with severe COVID-19 (Söndergaard, 2021) is illustrative of how an explanatory framework may guide policy recommendation.

## Implications for public health policy

Why then is it important to pay attention to the explanatory frameworks that surround the debate on ethnic health disparities? As indicated throughout the text, explanatory frameworks matter as they guide policy interventions and strategies. In its simplest form, it may be that socioeconomic explanations lead us to think of interventions focusing on combating poverty, improving housing and work conditions, whereas a culturalist framework tends to focus our attention on group identity and group behaviors, and a biological framework would seek biomedical solutions. However, reality is rarely this simple. Rather, complexity and contradictions are to be expected. From techno-science studies we know that medical practices are infused with contradictions and inconsistencies, and that “objects” are frequently assigned different ontologies by different actors (Mol, 2002; Shim, 2002). As such, “ethnic health disparities” may figure as a “boundary object,” stable enough to remain intact and recognizable by different stakeholders, yet interpreted differently in different contexts. As a final comment we would like to point to two COVID-19 policy initiatives that illustrate this complexity: that of the treatment of pregnant women with confirmed COVID-19 infections, and that of prioritization in the COVID-19 vaccination.

The policy targeting the treatment of pregnant women with confirmed COVID-19 infections is a regional policy from the southern-most part of Sweden issued in the autumn of 2020 (Region Skåne, 2020). Among other things, it addresses the increased risk of thromboembolism that comes with severe COVID-19 as well as with pregnancy. The policy document mentions both ethnicity (“with background in Africa, the Middle East or South Asia”) and socio-economics as at-risk indicators. Importantly, it suggests that women of such backgrounds should routinely be offered prophylaxis because of their alleged higher risk of severe COVID-19; for example, COVID-19 positive women considered having an African background should be given a prophylactic drug whereas COVID-19 positive “Swedish” women should not. The document does not provide any more in-depth evidence as to why certain groups of women are more susceptible to severe COVID-19, but while ethnicity in this context may refer to a presumed biological susceptibility, socio-economics most likely does not.

Concerning the second policy, the COVID-19 vaccine roll-out, the Public Health Agency initially put forward that (in addition to age and certain comorbidities) “socioeconomics, country of birth and residential area” are connected to enhanced risk for serious illness and death, and therefore should be taken into account when prioritizing patients (Folkhälsomyndigheten, 2021). Specifically, the Agency suggested that people living in socially vulnerable situations should be considered a prioritized group. Notably, in their initial report, the Agency highlighted “undocumented migrants” as an example of a socially vulnerable group. This caused strong objections from center-right wing pundits and politicians who considered it a disgrace that undocumented migrants should be prioritized over Swedish citizens (see e.g. *Göteborgsposten*, 2021). The Agency subsequently withdrew the list, but the category “socially vulnerable groups” remains.

Both cases are thus telling examples of how knowledge produced within a socioeconomic framework may nevertheless be translated into different kinds of biomedical interventions. In the case of vaccine prioritization, however, the suggestion was blocked by actors that had a different agenda and that perceived the ethnic health disparity through a culturalist framework. This complexity calls for a more in-depth scrutiny that does not only identify the existing dominant discourses, but also explores how they may be coordinated and translated in different ways and with different outcomes.

As regards COVID-19, ethnic health disparities in relation to COVID-19 remain an urgent public health issue. Nearly 2 years into the pandemic, statistics showed that people with “foreign background” continued to be identified as most affected by the pandemic. Whereas age was the main risk factor for the entire population, in the younger cohorts – below the age of 65 – mortality rates revealed that over 50% had foreign backgrounds (Socialstyrelsen, 2020). How to curb this development continues to be as acute as it was during the first wave.
